# Prevalence of extended spectrum beta lactamase producing Escherichia coli and Klebsiella pneumoniae urinary isolates in a tertiary care hospital in Kathmandu, Nepal

**DOI:** 10.1186/1756-0500-6-487

**Published:** 2013-11-25

**Authors:** Anil Chander, Chandrika Devi Shrestha

**Affiliations:** 1 Department of Microbiology, Kathmandu Medical College Teaching Hospital Sinamangal / Duwakot, Kathmandu, Nepal

**Keywords:** Antimicrobial resistance, Extended spectrum beta lactamase (ESBL), *Escherichia coli*, *Klebsiella pneumoniae*, Mid stream urine (MSU), Prevalence, Urinary isolates

## Abstract

**Background:**

*Escherichia coli* and *Klebsiella pneumoniae* are the major bacterial pathogens being isolated and reported from mid stream urine (MSU) specimens, globally. These uropathogens are mostly implicated as the major extended spectrum beta-lactamase (ESBL) producers, severely limiting the therapeutic management in cases of urinary tract infections. Limited studies had been reported from Nepal investigating the ESBL producers among uropathogens. This study was designed to assess the prevalence of ESBL producing *E.coli* and *K. pneumoniae* in urinary isolates at a centrally located major tertiary care hospital in Kathmandu valley, Nepal.

**Methods:**

Between September 2011 and May 2012, during the nine months period, 6308 MSU specimens were collected aseptically from the same number of clinically suspected patients of urinary tract infections. The samples were cultured on MacConkey agar and blood agar. The isolates with significant bacteriuria (10^5^ CFU / ml) were identified based on standard laboratory procedures. Antimicrobial susceptibility tests were carried out using various antimicrobial discs alongwith ceftriaxone on *E.coli* and *K. pneumoniae* isolates by Kirby Bauer disc diffusion method as per the recommendations of CLSI. On initial screening with ceftriaxone (30 μg) disc showing resistance was then confirmed for ESBL production by phenotypic confirmatory disc diffusion test (PCDDT) using ceftazidime (30 ug) and ceftazidime + clavulanic acid (30 μg + 10ug) disc as per guidelines of CLSI (2011).

**Results:**

Out of a total of 6308 MSU specimens investigated for significant bacteriuria, *E.coli* isolates were 444 (7.04%) and *K.pneuminiae* were 145 (2.3%) making a total of 589 (9.34%). Initial screening with ceftriaxone disc revealed 155 isolates of *E.coli* and 70 isolates of *K.pneumoniae* to be resistant. Further testing by PCDDT method showed 60/444 (=13.51%) of *E. coli* and 24/145 (=16.55%) of *K. pneumoniae* isolates to be confirmed ESBL producers. These ESBL – producer uropathogens showed high degree of resistance to ceftriaxone (100.0%), amoxycillin, fluoroquinolones and co-trimoxazole.

**Conclusion:**

An emerging and moderately high prevalence of ESBL-producing *E. coli* and *K. pneumoniae* was observed and confirmed in the urinary isolates investigated. It is essential to have a regular and routine monitoring of ESBL producing clinical isolates in laboratory practice.

## Background

Antimicrobial resistance among bacterial strains is an emerging problem, worldwide. Urinary tract infections (UTIs) are one of the most common bacterial infections in humans both in the community and the hospital settings [1-3]. *Escherichia coli* and *Klebsiella pneumoniae* are the two pre-dominant pathogens commonly isolated in urine. These uropathogens have also developed resistance to commonly prescribed antimicrobial agents, This severely limits the treatment options of an effective therapy.

Primarily, these uropathogens exerts their antimicrobial resistance against beta-lactams by producing extended spectrum beta-lactamases (ESBLs) enzymes that confers bacterial resistance to all beta-lactams except carbapenems, cephamycins and clavulanic acid [4-6]. ESBLs are class A beta-lactamases, and are plasmid-mediated enzymes that hydrolyze oxyimino-cephalosporins and monobactams, with various genotypes such as SHV, TEM, CTX-M, VEB, PER, BEL-1, BES-1, TLA and IBC [7,8]. Further, plasmids carrying ESBLs are transferable from one bacterial strain to the next and between different bacterial species [8]. ESBLs are clinically significant and when detected, indicate the need for the use of appropriate antibacterial agents. The mortality rate in misdiagnosed patients with ESBLs producing UTIs have ranged from 42-100% [9-11]. Antibacterial choice is often complicated by multi-resistance. There is an increasing association between ESBL production and fluoroquinolone resistance [12,13] and aminoglycoside resistance [14]. Multi-drug resistance (MDR) among ESBL expressing strains is complex and is influenced by the location of resistance genes on integrons that possess promoters that drive the co-ordinated expression of downstream resistance cassettes [15]. Thus, multi-resistance has severely limited the treatment options for ESBLs producing strains of Enterobacteriaceae. More recently, the emergence of carbapenem resistance has been reported among ESBL producing organisms [8].

The prevalence of bacteria producing ESBLs varies world-wide, with reports from North America, Europe, South America, Africa and Asia [16-18]. There is ample evidence to suggest the spread of ESBL infections is higher in resource poor countries [19,20].

Limited number of studies had been reported in this regard from Nepal [21-23]. This study was thus designed to estimate the current prevalence and antimicrobial resistance patterns among ESBL producing urinary isolates of *E.coli* and *K.pneumoniae* in a centrally located tertiary care centre in Kathmandu valley, Nepal.

## Methods

### Setting

This investigation was carried out in the Department of Microbiology, Kathmandu Medical College Teaching Hospital (affiliate Kathmandu University), a tertiary healthcare facility centrally located in the capital city of Kathmandu, Nepal. Ethical approval was not required to carry out this work as the bacterial isolates were collected as part of standard patient care investigation.

### Specimens

Between 1st September 2011 and 31st May 2012, 6308 consecutive and non-duplicate mid-stream urine specimens were collected aseptically from clinically suspected patients of UTIs attending outpatient and inpatient departments (medicine, surgery, paediatrics and gynecology and obstetrics) of the hospital. Urine specimens were transported to the Department of Microbiology laboratory and processed immediately.

### Inclusion criteria

Patients who did not have a course of antibiotics at least two weeks prior to selection in the study.

### Identification of isolates

The urine sample was cultured on to MacConkey agar and blood agar plates and incubated at 37°C for 24 hours and the colonies identified based on morphology and gram negative bacilli isolated were characterized by performing gram’s staining, motility and standard biochemical tests [24].

### Anti-microbial susceptibility testing

*E.coli* and *K. pneumoniae* isolates were selected to determine their susceptibility patterns against the first line antimicrobial agents, by the disc diffusion method of Kirby- Bauer as described by Clinical Laboratory Standards Institute (CLSI) [25]. The antimicrobial agents used were: amikacin (30 μg), gentamicin (10 μg), ofloxacin (5 μg), norfloxacin (10 μg), nitrofurantoin (300 μg), amoxicillin (10 μg), nalidixic acid (30 μg), co-trimoxazole (1.25/23.75 μg) and ceftriaxone (30 μg).

### Screening of ESBL producing strains of *E.coli* and *K.pneumoniae*

Strains showing zone of inhibition of ≤ 25 mm for ceftriaxone were selected for confirmation test of ESBL, according to guidelines of CLSI [25].

### Phenotypic Confirmatory Disc Diffusion Test (PCDDT) for ESBL

The potential ESBL producing strains of *E.coli* and *K.pneumoniae* were confirmed for ESBL production by phenotypic test. Briefly, a lawn culture of the isolated bacteria on Mueller Hinton agar (MHA) was made and ceftazidime (30 μg) and the combination disc ceftazidime + clavulanic acid (30 μg + 10 μg) was placed with 25 mm apart. An increase of ≥ 5 mm in zone of inhibition for ceftazidime + clavulanic acid compared to ceftazidime alone was confirmed as ESBL producing strains, as per recommendations of CLSI [25]. ESBL positive strains of *E.coli* and *K. pneumoniae* were selected to determine their susceptibility patterns against the second line of antimicrobial agent (imipenem −10 μg), by the disc diffusion method of Kirby- Bauer as described by the CLSI [25]. The antimicrobial agents disc were obtained from Hi-Media Laboratories Pvt. Ltd., Mumbai, India. *E.coli* ATCC 25922 (ESBL negative) and *K.pneumoniae* ATCC 700603 (ESBL positive) were used as controls throughout the study.

All data were analyzed using SPSS statistical software (SPSS Inc., Chicago, IL).

## Results

Out of a total of 6308 mid-stream urine specimens screened for significant bacteriuria, a total of 444 (7.03%) and 145 (2.29%) isolates of *E.coli* and *K.pneumoniae* were confirmed, respectively. Initial screening of these isolates for ESBL production showed 155/ 444 of *E.coli* and 70/145 of *K.pneumoniae* strains to be ceftriaxone resistant. Confirmation test (PCDDT) revealed 60/444 (13.51%) of *E.coli* and 24/145 (16.55%) of *K.pneumoniae* isolates to be ESBL positive (Table 1). A high rate of non-ESBL mediated ceftriaxone resistance was observed i.e. (95/155 = 63.33%) for *E.coli* and (46/70 = 65.71%) for *K.pneumoniae* isolates.

**Table 1:** **ESBL pattern of *E.coli *** **and *Klebsiella pneumoniae *** **isolated from urine specimens**

**Bacterial sp. isolated**	**No. of isolates obtained / total bacterial specimens screened (%)**	**No. of screened and selected isolates for ESBL confirmatory test (ceftriaxone resistant)**	**No. of isolates confirmed by PCDDT / No. of isolates obtained (%)**
*E.coli*	444/6308 (7.03)	155	60 /444 (13.51)
*K.pneumoniae*	145/6308 (2.29)	70	24 /145 (16.55)

The age varied from 7 months to 72 years and the sex ratio male:female was (1:2), of the patients from whom *E.coli* was isolated. The age range was 17 years to 70 years and the sex ratio male:female was (1:2.4) of the patients from whom *K.pneumoniae* was isolated. Clearly, the female group constituted the majority of the patients (Table 2).

**Table 2:** **Age groups and sex-wise details of patients with ESBL positive *E.coli *** **and ESBL positive *K.pneumoniae *** **isolates**

**Age group (years)**	**ESBL positive *E.coli***			**ESBL positive *K.pneumoniae***		
	**M**	**F**	**Total**	**M**	**F**	**Total**
< 15	6	2	8	0	0	0
16-30	2	20	22	2	10	12
31-45	8	10	18	1	1	2
46-60	2	2	4	2	2	4
>60	6	2	8	4	2	6
Total	24	36	60	9	15	24

A wide spectrum of resistance patterns to various antimicrobial agents was shown by the ESBL positive *E.coli* and *K.pneumoniae* strains. These *E.coli* strains showed a high degree of resistance (> 80%-95%) to ofloxacin, norfloxacin, co-trimoxazole, nalidixic acid and amoxicillin. The *E.coli* strains were much less resistant to gentamicin (23.33%), nitrofurantoin (11.66%) and least resistant to amikacin (6.66%).

*K.pneumoniae* isolates were maximally resistant (100%) to amoxicillin. A moderately high resistance of 66.66%, 58.33% and 45.83% were shown to nalidixic acid, co-trimoxazole and gentamicin, respectively by these *K. pneumoniae* strains. Moderate resistance of 37.5% and 29.16% were shown toward the fluoroquinolone drugs norfloxacin and ofloxacin. Nitrofurantoin (20.83% resistantance) and amikacin (12.5% resistance) were the most effective antimicrobial agents against ESBL producing *K.pneumoniae* isolates. All the *E.coli* and *K.pneumoniae* isolates were susceptible (100%) to the carbapenem, imipenem (Table 3).

**Table 3:** **Antimicrobial resistance patterns of confirmed ESBL producing *E.coli *** **and *K.pneumoniae *** **isolates from urine specimens**

**Antibiotic**	**ESBL positive *E.coli *** **(n = 60)**	**ESBL positive *K.pneumoniae *** **(n = 24)**
	**T/ R (%)**	**T/R (%)**
Amikacin	60/04 (6.66)	24/3 (12.5)
Gentamicin	60/14 (23.33)	24/11 (45.83)
Ofloxacin	60/49 (81.66)	24/07 (29.16)
Norfloxacin	60/54 (90.0)	24/9 (37.5)
Nitrofurantoin	60/07 (11.66)	24/05 (20.83)
Amoxycillin	60/57 (95.0)	24/24 (100.0)
Nalidixic acid	60/57 (95.0)	24/16 (66.66)
Co-trimoxazole	60/51 (85.0)	24/14 (58.33)
Ceftriaxone	60/60 (100.0)	24/24 (100.0)
Ceftazidime	60/60 (100.0)	24/24 (100.0)
Imipenem	60/0 (0.0)	24/0 (0.0)

T = Total no. of isolates tested; R = No. of isolates resistant.

A high multi-drug resistance (defined as resistant to ≥ 3 different classes of antimicrobial agents) of 91.66% and 87.5% respectively was observed among the ESBL producing strains of *E.coli* and *K.pneumoniae*. None of the strains of ESBL positive *E.coli* and *K.pneumoniae* were found to be sensitive to all the antimicrobial agents tested (Table 4).

**Table 4:** **Antimicrobial resistance patterns among ESBL positive *E.coli *** **and ESBL positive *K.pneumoniae *** **urine isolates**

**ESBL positive *E.coli *** **isolates no. (%)**	**ESBL positive *K.pneumoniae *** **isolates no. (%)**	**Resistance to no. of classes of antibiotics tested**
0	0	0
0	0	1
5 (8.34)	3 (12.5)	2
55(91.66)	21 (87.5)	≥3

## Discussion

There is a paucity of documented literature on investigation of ESBLs producing uropathogens from Nepal [23]. To the best of our knowledge this is the first report from Nepal with an exclusive focus on investigating the current prevalence and antimicrobial susceptibility patterns among ESBL producing *E. coli* and *K. pneumoniae* – the predominant uropathogens, globally. The urine culture positivity rate was 9.32% (*E.coli* =7.03% and *K.pneumoniae* = 2.29%), which was low compared to that reported from South Africa (51%) [26]. Nicaragua (30%) [27] and India (39%) [28]. ESBL production was more prevalent in *K.pneumoniae* strains, as compared to that in *E.coli* strains, which agrees with findings reported in previous studies [13,29]. The occurrence of ESBLs among clinical isolates vary greatly worldwide and geographically and are rapidly changing overtime. ESBL prevalence was 29.86% (*E.coli* = 13.51% and *K.pneumoniae* = 16.55%). It was low as compared to a study done in India [30], which reported nearly 40% of urinary isolates of *E.coli* and *K.pneumoniae* were ESBL positive. The highest isolation rate of ESBLs producing *K.pneumoniae* had been reported from the Latin America (54.4%), the western Pacific (24.6%) and Europe (22.6%). The frequency of ESBL producing *E.coli* in these areas was reported to be 8.5%, 7.8% and 5.3%, respectively [31]. Females showed a higher rate of isolation of ESBL producing *E.coli* (60%) and *K.pneumoniae* (62.5%), which parallels the findings as reported earlier [32,33]. This study revealed a higher occurrence of ESBL producing uropathogens in the adult age group of 16–45 years, which is similar to that reported in a study done in Pakistan [1].

The high rates for non – ESBL mediated ceftriaxone resistant *E.coli* (63.33%) and *K.pneumoniae* (65.71%) isolates may be due to their different mechanisms for resistance such as the production of ampC β lactamase, metallo-betalactamase, etc. [34]. This further limits the therapeutic options available to treat these infections.

The susceptibility patterns of the ESBL producing *E.coli* and *K. pneumoniae* varied widely with the class of antimicrobial agents used. All isolates were uniformly resistant (100%) to the third generation cephalosporin, ceftriaxone. All ESBL positive *K.pneumoniae* strains were resistant (100%) to amoxicillin, while resistance rate for ESBL positive *E.coli* was 95%. In a study reported from Madagascar [35], 80%of the *E.coli* isolates were resistant to amoxicillin. In our study *E.coli* showed a high resistance rate (85%) to co-trimoxazole where as the *K.pneumoniae* showed resistance rate (58.33%) to co-trimoxazole. A comparable resistance rate of 80% and 45% to co-trimoxazole was shown among ESBL producing *E.coli* and *K.pneumoniae* isolates in a study conducted in Iran [36]. A striking feature in this study was that the quinolones, ofloxacin, norfloxacin and nalidixic acid demonstrated a high resistance varying from > 80% to 95% among ESBL positive *E.coli*, while 29.16% to 66.66% of *K. pneumoniae* were resistant. This parallels with the findings of the studies done in Indore, India [37] and Bangladesh [38]. A lower resistance rates varying from 24% to 44% to norfloxacin had been reported in European countries [39]. This probably reflects a better management of these clinically significant infections in resource-rich countries.

Conversely, this study revealed resistance rates of 45.83% and 12.5% to the aminoglycosides, gentamicin and amikacin by ESBL positive *K.pneumoniae* strains. In comparison, *E.coli* shown a much low resistance rate - gentamicin (23.33%) and amikacin (6.66%). In contrast. *K. pneumoniae* showed resistance to gentamicin (69%) and amikacin (38%) while 59% and 33% resistance rates were shown by *E.coli* isolates in a study done in Indore, India [37]. A resistance rate of 46.7% to gentamicin was shown by *K.pneumoniae* isolates in a report from Karachi, Pakistan [40], which is similar to results in this study. This report supports that aminoglycosides have good activity against clinically important gram negative bacilli [41]. The resistance rates of ESBL positive *E.coli* and *K.pneumoniae* to nitrofurantoin were 11.66% and 20.83%, respectively. Parallel resistance rate (12%) by *E.coli* isolates was shown in a report from Indore, India [37].

A significant finding in this report was that aminoglycosides and nitrofurantoin proved to be the optimal drugs. This may be due to the restricted use of these drugs in our hospital setting and nitrofurantoin is usually reserved to be prescribed only in cases of UTIs since it is excreted and concentrated in urine. All ESBL positive *E.coli* and *K.pneumoniae* strains in this study were sensitive (100%) to imipenem. A very high MDR rates of 91.66% and 87.5% among ESBL positive *E.coli* and *K.pneumoniae* were obtained in this study. A comparable MDR rate of 83% among *E.coli* isolates was reported in a study done in Peshawar, Pakistan [1].

Nepal represents a unique geographical terrain with sparsely-populated areas mainly in hilly and mountainous regions, severely limited health-care resources and a low-income economy. And the health care facilities are concentrated in the capital city of Kathmandu and people come here for treatment, from far-flung areas of the country.These people may acquire pathogens and when they return to their native areas in villages, may act as carriers of drug resistant pathogens.

Multi-center studies involving major health-care facilities in other parts of the country are required to have a more clear picture of ESBL producing uropathogens. Further, molecular epidemiological studies of resistance genes among the uropathogens would provide us much needed details on bacterial clones circulating in this region.

## Conclusion

This report documents the emergence and occurrence of ESBL producing *E.coli* and *K.pneumoniae* in urinary isolates in Nepal. A moderately high prevalence of ESBL producing *E.coli* and *K.pneumoniae* was observed and confirmed in the urinary isolates. A strict hospital infection control policies and a prudent anti-microbials use regimens are to be adopted by the physicians. It is essential and mandatory to have a regular and routine monitoring of ESBL producing clinical isolates in clinical laboratories.

## Abbreviations

MSU: Mid stream urine; ESBL: Extended spectrum beta-lactamase; NCCLS: National committee on clinical laboratory standards; PCDDT: Phenotypic confirmatory disk diffusion test; CLSI: Clinical laboratory standards institute; UTIs: Urinary tract infections; MHA: Mueller Hinton Agar; MDR: Multi drug resistance.

## Competing interests

The authors declare that they have no competing interests.

## Authors’ contributions

AC did the interpretation, statistical analysis of data and drafted the manuscript. CDS has been involved in conception, design and acquisition of data. Both authors have read and approved the final manuscript.

## References

[B1] Ullah F Malik SA Ahmed J Antibiotic susceptibility pattern and ESBL prevalence in nosocomial Escherichia coli from urinary tract infections in Pakistan Afr J Biotechnol 2009 8 16 3921 3926

[B2] Cox CE Nosocomial urinary tract infections Urol 1988 32 3 210 215 10.1016/0090-4295(88)90386-X 3046098

[B3] Gonzalez CM Schaeffer AJ Treatment of urinary tract infection: what’s old, what’s new, and what works World J Urol 1999 17 6 372 382 10.1007/s003450050163 10654368

[B4] Coque TM Baquero F Canton R Increasing prevalence of ESBL producing enterobacteriaceae in Europe Eurosurveillance 2008 13 47 1 11 19021958

[B5] Paterson DL Bonomo RA Extended spectrum β-lactamases: a clinical update Clin Microbiol Rev 2005 18 4 657 686 10.1128/CMR.18.4.657-686.2005 16223952 PMC1265908

[B6] Bradford PA Extended spectrum β-lactamases in the 21st century: characterization, epidemiology, and detection of this important resistance threat Clin Microbiol Rev 2001 14 4 933 951 10.1128/CMR.14.4.933-951.2001 11585791 PMC89009

[B7] Jacoby GA Munoz-Price LS The new beta-lactamases New Engl J Med 2005 352 4 380 391 10.1056/NEJMra041359 15673804

[B8] Rupp ME Fey PD Extended spectrum β-lactamase (ESBL)- producing enterobacteriaceae: considerations for diagnosis, prevention and drug treatment Drugs 2003 63 4 353 365 10.2165/00003495-200363040-00002 12558458

[B9] Meyer KS Urban C Eagan JA Berger BJ Rahal JJ Nosocomial outbeak of *Klebsiella* infection resistant to late-generation cephalosporins Ann Intern Med 1993 119 353 358 10.7326/0003-4819-119-5-199309010-00001 8135915

[B10] Naumovsky L Quinn JP Miyashiro D Patel M Bush K Singer SB Outbreak of ceftazidime resistance due to a novel extended spectrum β-lactamase in isolates from cancer patients Antimicrob Agents Chemother 1992 36 1991 1996 10.1128/AAC.36.9.1991 1416892 PMC192997

[B11] Schiappa DA Hayden MK Matushek MG Hashemi FN Sullivan J Smith KY Ceftazidime resistant *Klebsiella pneumoniae* and *Escherichia* coli blood stream infection: a case control and molecular epidemiologic investigation J Infect Dis 1996 174 529 536 10.1093/infdis/174.3.529 8769610

[B12] Paterson DL Mulazimoglu L Casellas JM Ko W-C Goossens H Gottberg AV Epidemiology of ciprofloxacin resistance and its relationship to extended spectrum β-lactamase production in *Klebsiella pneumoniae* isolates causing bacteremia Clin Infect Dis 2000 30 473 478 10.1086/313719 10722430

[B13] Spanu T Luzzaro F Perilli M Amicosante G Toniolo A Fadda G The Italian ESBL Study Group. Occurrence of extended spectrum β-lactamases in members of the family Enterobacteriaceae in Italy: implications for resistance to β-lactams and other antimicrobial drugs Antimicrob Agents Chemother 2002 46 196 202 10.1128/AAC.46.1.196-202.2002 11751134 PMC126983

[B14] Jacoby GA Sutton L Properties of plasmids responsible for production of extended spectrum β-lactamases Antimicrob Agents Chemother 1991 35 164 169 10.1128/AAC.35.1.164 1849707 PMC244959

[B15] Poirel M Le Thomas I Naas T Karim A Nordmann P Biochemical sequence analyses of GES-1, a novel class A extended spectrum β-lactamase, and the Class I integrin In52 from *Klebsiella pneumoniae* Antimicrob Agents Chemother 2000 44 622 632 10.1128/AAC.44.3.622-632.2000 10681329 PMC89737

[B16] Bonnet R Growing group of extended spectrum β-lactamases: the CTX-M enzymes Antimicrob Agents Chemother 2004 48 1 1 14 10.1128/AAC.48.1.1-14.2004 14693512 PMC310187

[B17] Falagas ME Karageorgopoulos Extended spectrum β-lactamase-producing organisms J Hosp Infect 2009 73 4 345 354 10.1016/j.jhin.2009.02.021 19596491

[B18] Reinert RR Low DE Rossi F Zhang X Wattal C Dowzicky MJ Antimicrobial susceptibility among organisms from the Asia/ Pacific Rim, Europe and Latin and North America collected as part of TEST and the in vitro activity of tigecycline J Antimicrob Chemother 2007 60 5 1018 1029 10.1093/jac/dkm310 17855724

[B19] Villegas MV Kattan JN Quinteros MG Casselas JM Prevalence of extended-spectrum β-lactamases in South America Clin Microbiol Infect 2008 14 Suppl. 1 154 158 18154539 10.1111/j.1469-0691.2007.01869.x

[B20] Sader HS Jones RN Gales AC Silva JB Pignatari AC SENTRY antimicrobial surveillance program report; Latin America and Brazilian results for 1997 through 2001 Braz J Infect Dis 2004 8 1 25 79 10.1590/S1413-86702004000100004 15286878

[B21] Poudyal S Bhatta DR Shakya G Upadhyaya B Dumre SP Buda G Extended spectrum β-lactamase producing multidrug resistant clinical bacterial isolates at National Public Health Laboratory, Nepal Nepal Med Coll J 2011 13 1 34 38 21991699

[B22] Shrestha S Amatya R Dutta R Prevalence of extended spectrum beta lactamase (ESBL) production in gram negative isolates from pyogenic infection in tertiary care hospital of Eastern Nepal Nepal Med Coll J 2011 13 3 186 189 22808812

[B23] Baral P Neupane S Marasini BP Ghimire KR Lekhak B Shrestha B High prevalence of multidrug resistance in bacterial uropathogens from Kathmandu, Nepal BMC Res Notes 2012 5 38 10.1186/1756-0500-5-38 22260454 PMC3296586

[B24] Collee JG Miles RS Watt B Collee JG, Fraser AG, Marmion BP, Simmons A Tests for identification of bacteria Mackie and McCartney Practical Medical Microbiology 1996 14 New York: Churchill Livingstone 131 149

[B25] Clinical Laboratory Standards Institute Performance standards for antimicrobial susceptibility testing; Twenty First informational supplement (M 100-S21) 2011 Wayne PA: Clinical and Laboratory Standards Institute

[B26] Habte TM Dube S Ismail N Hoosen AA Hospital and community isolates of uropathogens at a tertiary hospital in South Africa S Afr Med J 2009 99 584 587 19908617

[B27] Matute AJ Hak E Schurink CAM McArthur A Alonso E Pantagua M Resistance of uropathogens in symptomatic urinary tract infections in Leon, Nicaragua Int J Antimicrob Agents 2004 23 506 509 10.1016/j.ijantimicag.2003.10.003 15120732

[B28] Tambekar DH Dhanorkar DV Gulhane DR Khanadelwal VK Dudhane MN Antibacterial susceptibility of some urinary tract pathogens to commonly used antibiotics Afr J Biotechnol 2006 5 1562 1565

[B29] Durmaz R Durmaz B Koroglu M Tekerekoglu MS Detection and typing of extended- spectrum β-lactamases in clinical isolates of the family Enterobacteriaceae in a medical center in Turkey Microb Drug Resist 2001 7 171 175 10.1089/10766290152045048 11442343

[B30] Babypadmini S Appalaraju B Extended spectrum lactamase in urinary isolates of *E.coli* and *Klebsiella pneumoniae* - prevalence and susceptibility patterns in a tertiary care hospital Indian J Med Microbiol 2004 22 172 174 17642726

[B31] Aminzadeh Z Sadat KM Sha’bani M Bacteriuria by extended spectrum beta-lactamase producing *E.coli* and *Klebsiella pneumoniae* isolates in a government hospital in south of Tehran, Iran Iran J Kidney Dis 2008 2 197 200 19377237

[B32] Ahmed SM Jakribettu RP Koyakutty S Arya B Shakir VPA Urinary tract infections – an overview on the prevalence and the anti-biogram of gram negative uropathogens in a tertiary care center in North Kerala, India J Clin Diagn Res 2012 6 1192 1195

[B33] Oladeinde BH Omoregie R Olley M Anunibe JE Urinary tract infections in a rural community of Nigeria North Amer J Med Sci 2011 3 75 77 10.4297/najms.2011.375 PMC3336890 22540069

[B34] Dalela G Gupta S Jain DK Mehta P Antibiotic resistance patterns in uropathogens at a tertiary care hospital at Jhalawar with special reference to ESBL, AmpC b-lactamase and MRSA production J Clin Diagn Res 2012 6 4 645 651

[B35] Randrianirina F Soares J-L Carod J-F Ratsima E Thonnier V Combe P Antimicrobial resistance among uropathogens that cause community-acquired urinary tract infections in Antananarivo, Madagascar J Antimicrob Chemother 2007 59 309 312 17138569 10.1093/jac/dkl466

[B36] Behrooozi A Rahbar M Yousefi JV Frequency of extended spectrum beta-lactamase (ESBLs) producing *Escherichia coli* and *Klebsiella pneumoniae* isolated from urine in an Iranian 1000-bed tertiary care hospital Afr J Microbiol Res 2010 4 9 881 884

[B37] Pathak A Marothi Y Kekre V Mahadik K Macaden R Lundborg CS High prevalence of extended spectrum β-lactamase producing pathogens: results of a surveillance study in two hospitals in Ujjain, India Infect Drug Resist 2012 5 65 73 22570555 10.2147/IDR.S30043 PMC3345881

[B38] Alipourfard I Nili NY Antibiogram of extended spectrum beta-lactamase (ESBLs) producing *Escherichia coli* and *Klebsiella pneumoniae* isolated from hospital samples Bangladesh J Med Microbiol 2010 4 1 32 36

[B39] van der Donk CFM van de Bovenkamp JHB De Brauwer EIGB De Mol P Feldhoff K-H Kalka-Moll WM Antimicrobial resistance and spread of multi drug resistant *Escherichia coli* isolates collected from nine urology services in the Euregion Meuse-Rhine PLoS One 2012 7 10 e47707 10.1371/journal.pone.0047707 23082197 PMC3474752

[B40] Abdullah FE Mushtaq A Irshad M Rauf H Afzal N Rasheed A Current efficacy of antibiotics against *Klebsiella* isolates from urine samples – a multi-centric experience in Karachi Pak J Pharm Sci 2013 26 1 11 15 23261722

[B41] Gonzalez LS Spencer JP Aminoglycosides: a practical review Am Fam Physician 1998 58 8 1811 1820 9835856

